# Bright monomeric fluorescent protein elite-niRFP704 for two-channel near-infrared STED nanoscopy

**DOI:** 10.1073/pnas.2533451123

**Published:** 2026-08-25

**Authors:** Florian Habenstein, Nickels Jensen, Daniel Stumpf, Alexey I. Chizhik, Marcel Leutenegger, Jin Chang, Andreas Fognini, Jessie Qin-Dregely, Iman Esmaeil Zadeh, Laura L. Kirck, Jörg Enderlein, Kaushik Inamdar, Stefan W. Hell, Stefan Jakobs

**Affiliations:** ^a^ Department of NanoBiophotonics, Max Planck Institute for Multidisciplinary Sciences, Göttingen 37077, Germany; ^b^III. Institute of Physics, Georg August University, Göttingen 37077, Germany; ^c^ Optics Research Group, ImPhys Department, Faculty of Applied Sciences, Delft University of Technology, Delft 2628 CJ, The Netherlands; ^d^Single Quantum B.V., Delft 2629 HH, The Netherlands; ^e^ Fraunhofer Institute for Translational Medicine and Pharmacology ITMP, Translational Neuroinflammation and Automated Microscopy TNM, Göttingen 37075, Germany; ^f^ Multiscale Bioimaging Cluster of Excellence, University of Göttingen, Göttingen 37075, Germany; ^g^ Clinic of Neurology, University of Göttingen, Göttingen 37075, Germany; ^h^ Department of Optical Nanoscopy, Max Planck Institute for Medical Research, Heidelberg 69120, Germany

**Keywords:** superresolution microscopy, STED, bacteriophytochrome, fluorescent protein, live-cell imaging

## Abstract

The near-infrared (NIR) spectral region is attractive for live-cell imaging, due to low autofluorescence and reduced phototoxicity. Some phytochrome-derived fluorescent proteins absorb and emit fluorescence in the NIR, but have short fluorescence lifetimes and relatively low quantum yields, requiring higher laser powers thus limiting their usefulness for live-cell superresolution microscopy. Using the bacterial phytochrome miRFP703 as a template, we screened for variants with longer fluorescence lifetimes, because the quantum yield and fluorescence lifetime are linked. We identified the bright monomeric fluorescent protein elite-niRFP704, which has a longer lifetime (1.12 ns) and a correspondingly higher quantum yield (0.21) than its template, absorbing and emitting completely in the NIR spectral region. elite-niRFP704 was used to tag proteins in living cells and facilitated extended stimulated emission depletion (STED) microscopy on cell lines stably expressing a fusion protein. Finally, elite-niRFP704 and miRFP703 could be separated based on their significantly different lifetimes, enabling two-channel NIR STED microscopy of living mammalian cells.

## Significance

Near-infrared (NIR) superresolution STED (stimulated emission depletion) microscopy in living cells is currently limited by a sparse selection of live-cell compatible bleaching-resistant fluorophores. To address this limitation, we screened variants of near-infrared bacterial phytochromes aiming for increasing fluorescence lifetimes, as this property is correlated with increased quantum yield. We identified elite-niRFP704, an NIR-compatible protein with a longer lifetime of 1.12 ns, increased quantum yield of 21% and thus higher molecular brightness over its template. Using elite-niRFP704 as a tag for cellular proteins, we performed STED imaging for up to 100 frames in living mammalian cells, while its extended fluorescence lifetime enabled lifetime-separation based two-channel NIR-STED imaging.

The red to near-infrared (NIR) spectral range between 650 and 900 nm wavelength is the preferred spectral region for live-cell imaging (1, 2). The autofluorescence stemming from abundant molecules such as nicotinamide adenine dinucleotide (NADH) and flavin adenine dinucleotide (FAD) is reduced in this spectral region compared to its near ultra-violet (UV) and visible spectral counterparts. Also, the absorbance of water, hemoglobin, and oxyhemoglobin reaches a minimum between 650 and 900 nm (3). Since many superresolution approaches such as STED nanoscopy rely on illumination patterns of relatively high light doses, the reduced scattering and the lower phototoxicity of NIR light is particularly beneficial to improve the live-cell and deep-tissue compatibility of these methods (4). Therefore, genetically encoded fluorescent labels absorbing and emitting in the NIR range are in high demand.

GFP-like fluorescent proteins are widely used genetically encoded fluorescent labels for live-cell imaging (5). However, they do not cover the >650 nm spectral excitation range. Thus, live-cell imaging with excitation and emission in the NIR region has been facilitated by the use of engineered phytochrome-based fluorescent proteins as genetically encoded fusion tags (6). Phytochromes are a family of light-sensing proteins found in plants, bacteria, and fungi. Most bacteriophytochromes consist of a photosensory core module (PCM), which includes a PAS (Per-ARNT-Sim) and a GAF (cGMP phosphodiesterase-adenylate cyclase-Fh1A) domain and a phytochrome-specific output (PHY) domain (7, 8). The external chromophore biliverdin (BV), which allows for absorption of light in the NIR region, is covalently bound via thioether linkage to a cysteine residue located in the GAF domain, while the chromophore-binding pocket is formed mainly by the PAS domain. Because BV is generated as a product of heme degradation (9), it is produced endogenously in mammalian cells. However, endogenous BV concentrations are typically insufficient to fully saturate chromophore binding to exogenously expressed bacteriophytochromes.

The first engineered bacteriophytochrome employed in fluorescence imaging, IFP1.4, was derived from the extremophile bacterium *Deinococcus radiodurans* (10). Since then, a variety of phytochrome-derived NIR fluorescent proteins have been reported (11–18).

All currently available bona fide bacteriophytochrome-derived NIR fluorescent proteins (excitation and emission >650 nm) exhibit a low fluorescence quantum yield below 15% (12, 14, 17, 18), a short fluorescence lifetime (below 1 ns) and a low molecular brightness compared to fluorescent proteins such as GFP (19), limiting their applicability in live-cell fluorescence microscopy and particularly in a superresolution STED (nanoscopy) modality. We previously developed the NIR absorbing fluorescent protein SNIFP (STED Near Infrared Fluorescent Protein), which represented the first reported phytochrome-based STED compatible NIR-FP. SNIFP, based on the *D. radiodurans* phytochrome, enabled live cell STED imaging of cellular structures, albeit for a limited duration (10 frames with a 60% loss in signal). While having a higher extinction coefficient, it still had a <1 ns lifetime and required relatively high STED laser powers (18, 20). Subsequently, a set of monomeric phytochrome-based NIR-FPs was developed, extending the achievable STED imaging duration to approximately 20 frames before signal intensity decreased by 50% (17). Some engineered bacterial phytochrome–based FPs such as miRFP670 (21), mIRFP663, and BDFP2 variants (22) exhibit increased quantum yields, but at the expense of a blue shift (23) so that their respective excitation maximum is no longer in the NIR-regime.

In this study, we took advantage of the well-documented correlation between fluorescence lifetime and fluorescence quantum yield in GFP-like fluorescent proteins (24–26). Previous screening based on fluorescence lifetime resulted in a set of fluorescent proteins targeting the visible spectrum that were used for lifetime-based separation in STED microscopy (27).

We established a lifetime-based screening strategy to increase the fluorescence quantum yield of a bacterial phytochrome template by directed and random mutagenesis. This approach led to the engineering of the NIR fluorescent protein elite-niRFP704, which is emitting and absorbing entirely in the NIR spectral region, while exhibiting a high fluorescence quantum yield (21%) and a comparatively long fluorescence lifetime (1.12 ns). Elite-niRFP704 was employed for live-cell STED superresolution microscopy with significantly extended bleaching resistance while requiring lower STED laser powers to achieve subdiffraction imaging. Its prolonged fluorescence lifetime enabled two-channel STED microscopy by fluorescence-lifetime imaging microscopy (FLIM) in the NIR spectral region using a single excitation laser.

## Results

### Fluorescence Lifetime Screening.

**Fig. 1.**
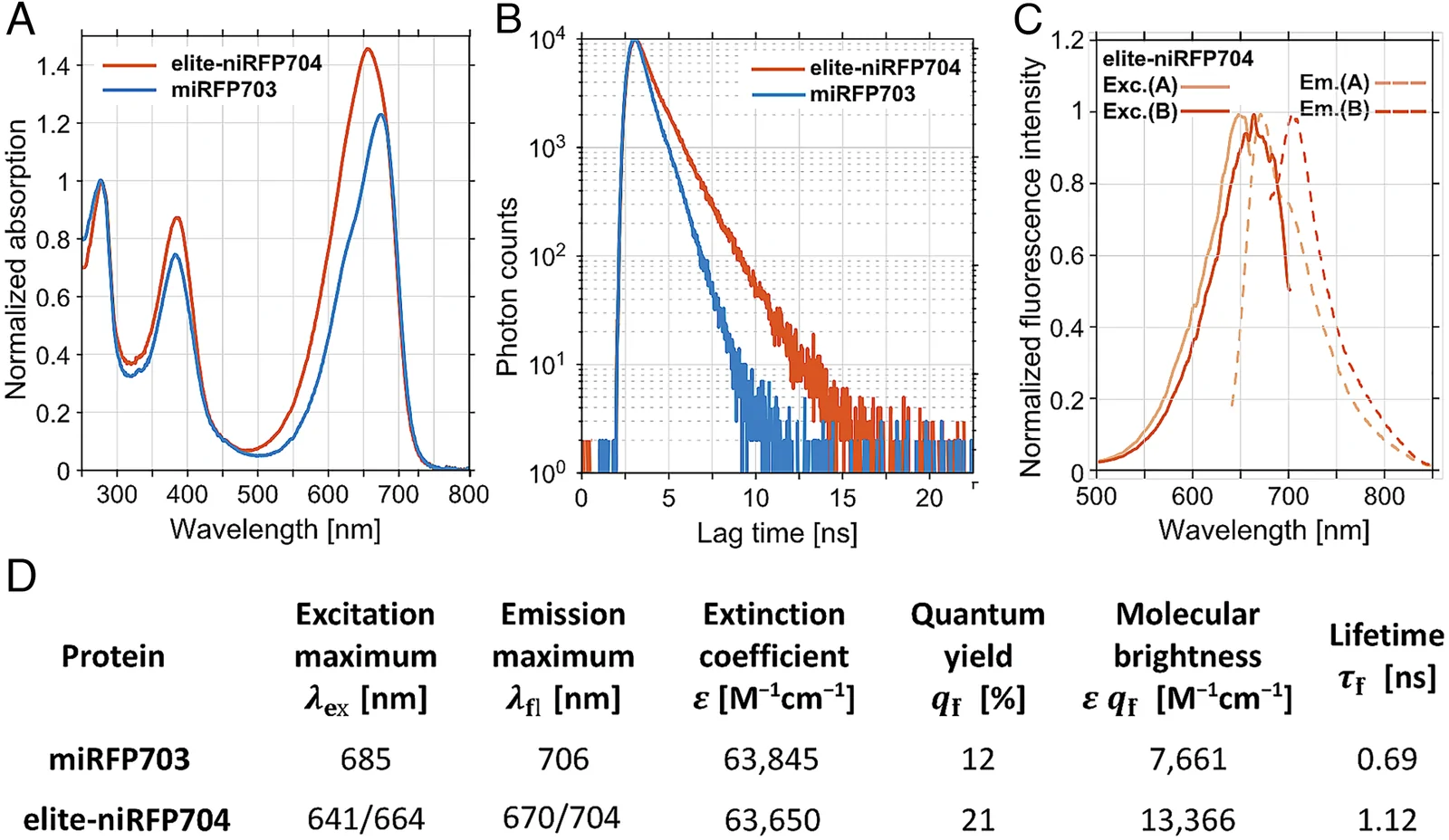
Spectral characterization and photophysical properties of elite-niRFP704 and its template miRFP703. (*A*) Absorption spectra normalized to the absorbance at 278 nm. (*B*) Fluorescence lifetime histograms; (*C*) Normalized excitation and fluorescence emission spectra of elite-niRFP704. Excitation spectrum A was recorded from 500 to 670 nm with fluorescence detection at 680 nm, whereas excitation spectrum B was recorded from 500 to 700 nm with fluorescence detection at 710 nm. Emission spectrum A was recorded from 640 to 850 nm with excitation at 630 nm, whereas emission spectrum B was recorded from 680 to 750 nm with excitation at 670 nm. (*D*) Photophysical properties.

We aimed at generating a phytochrome-based fluorescent protein absorbing and emitting in the NIR spectral range (650 to 900 nm), exhibiting a longer fluorescence lifetime and an increased fluorescence quantum yield. In order to screen for such an improved variant, the bacterial phytochrome miRFP703 was chosen as a template. miRFP703 is a NIR fluorescent protein that has been engineered from the bacterial phytochrome RpBphP1 of *Rhodopseudomonas palustris* (21). miRFP703 is a 34.5 kDa protein consisting of a PAS and a GAF domain. It is among the brightest monomeric NIR fluorescent proteins with absorption (Fig. 1*A*) as well as excitation and emission maxima [*SI Appendix*, Fig. S1*A*; (21)] in the NIR spectrum. Still, compared to GFP-like fluorescent proteins it features a low quantum yield of 8.6% and a short fluorescence lifetime of 0.69 ns (Fig. 1 *B* and *D*).

Based on crystal structures of miRFP703 and related proteins (PDB 5VIK, 5VIV) (28), we performed error-prone mutagenesis and site-directed mutagenesis at positions corresponding to amino acid positions in close proximity to the chromophore to create several bacterial expression plasmid libraries (see *Methods* for complete list of residue positions). These libraries were transformed into *Escherichia coli* cells and individual colonies were screened for expression of a variant with an increased fluorescence lifetime. To this end, a custom-built automated microscope equipped with a pulsed NIR laser system and an APD detector with TCSPC (time correlated single photon counting) electronics for time-resolved fluorescence detection was utilized to screen for bacterial colonies expressing proteins with longer fluorescence lifetime and higher brightness. Overall, about 10,000 different variants expressing single mutations were screened. In this screen, the mutation I253S resulted in an increased fluorescence lifetime (0.92 ns) and a higher quantum yield (18%). However, the *E. coli* BL21 AI cells expressing miRFP703-I253S did not show an increased cellular brightness, pointing to reduced expression levels, poor folding of this variant or impaired chromophore binding. To address this issue, several rounds of random mutagenesis on miRFP703-I253S and subsequent screening in bacterial cells were performed. Three additional mutations increased the effective brightness of the protein in the *E. coli* cells about threefold compared to miRFP703-I253S and increased the fluorescence lifetime further.

The resulting final variant thus had four mutations, namely F193L, V202C, Y210H, and I253S. We denote miRFP703-F193L, V202C, Y210H, I253S as elite-niRFP704 (extended-lifetime near-infrared fluorescent protein with an emission maximum at 704 nm) (Fig. 1 *C* and *D*). In elite-niRFP704, the fluorescence lifetime was increased from 0.69 ns in miRFP703 to 1.12 ns (Fig. 1 *B* and *D*). In order to determine fluorescence quantum yields, we relied on a plasmonic nanocavity (29). The plasmonic nanocavity approach provides an absolute measurement of the quantum yield that is based on tuning and measuring the fluorophore’s excited state lifetime. Hence, in contrast to quantum yield measurements based on the comparison to a known reference sample or on an integrating sphere, the measurement is not influenced by differences in the dark state population, incorrect folding, or the BV binding. Therefore, the quantum yields of both elite-niRFP704 and its template protein reported in this manuscript were determined with a plasmonic nanocavity. Using the absolute measurement approach, we consistently measured higher fluorescence quantum yields than previously reported with other methods. elite-niRFP704 exhibited a quantum yield of 21% compared to 12% of miRFP703 (Fig. 1*D*). With its fluorescence lifetime of 1.12 ns and fluorescence quantum yield of 21%, elite-niRFP704 has one of the longest lifetimes and highest quantum yields among all NIR-excited bacterial phytochromes reported so far.

### Properties of Elite-niRFP704.

Size exclusion chromatography confirmed that elite-niRFP704 exists as a monomer, similar to its template protein miRFP703 (*SI Appendix*, Fig. S1 *B* and *C*).

Protein stability was assessed over a pH range of 3 to 9, and the resulting fluorescence intensity profiles were fitted using a monophasic model (*SI Appendix*, Fig. S1*E*). A pKa value of 4.3 was determined, indicating that elite-niRFP704 is suitable for use as a fluorescent fusion tag in most mammalian cellular compartments. This finding was further supported by full absorption and emission spectra recorded across the tested pH range (*SI Appendix*, Fig. S1 *F*–*H*).

For elite-niRFP704, an extinction coefficient of 63,650 M^–1^ cm^–1^ at the absorption maximum (655 nm) was determined for the BV-bound holoprotein using the absorption maximum of the Soret band of the denatured protein as a reference [*SI Appendix*, Fig. S1*D*; (10)]. Applying the same experimental approach to miRFP703 yielded an extinction coefficient of 63,845 M^–1^cm^–1^, which differs from the previously reported value of 90,900 M^–1^ cm^–1^ (21). Consequently, elite-niRFP704 exhibited a molecular brightness of 13,366, corresponding to an approximately twofold increase compared to its template protein miRFP703 (Fig. 1*D*).

elite-niRFP704 exhibits an absorption maximum at 655 nm and additional absorption peaks at 278 nm, arising from aromatic amino acids, and at 388 nm, corresponding to the Soret band of biliverdin absorption (Fig. 1*A*). Compared with its template protein miRFP703, the Q-band absorption profile of elite-niRFP704 is broader and blue-shifted (Fig. 1*A*), suggesting alterations in its spectral properties. Furthermore, emission spectra recorded under blue-shifted (excitation at 630 nm; emission >640 nm) and red-shifted (excitation at 670 nm; emission >680 nm) excitation conditions revealed distinct emission maxima at 670 nm and 704 nm, respectively (Fig. 1*C*), indicating spectral heterogeneity within the fluorescent BV-bound population.

To further investigate the presence of distinct spectral states, excitation spectra were recorded at fixed emission wavelengths of 680 nm and 710 nm. The normalized excitation spectra exhibited two nonsuperimposable peaks with maxima at 641 nm and 664 nm, respectively, providing further evidence for the presence of multiple spectrally distinct states (Fig. 1*C*). Notably, the red-shifted excitation spectrum closely resembled the spectral characteristics of the template protein miRFP703 [*SI Appendix*, Fig. S1*A*, (21)], suggesting that the red-shifted population retains spectral properties similar to the parental fluorophore. Altogether, the excitation and emission maxima of the blue-shifted state were observed at 641 nm and 670 nm, respectively, whereas the excitation and emission maxima of the red-shifted state were observed at 664 nm and 704 nm, respectively (Fig. 1 *C* and *D*).

### Targeting of Elite-niRFP704 to Various Cellular Compartments.

**Fig. 2.**
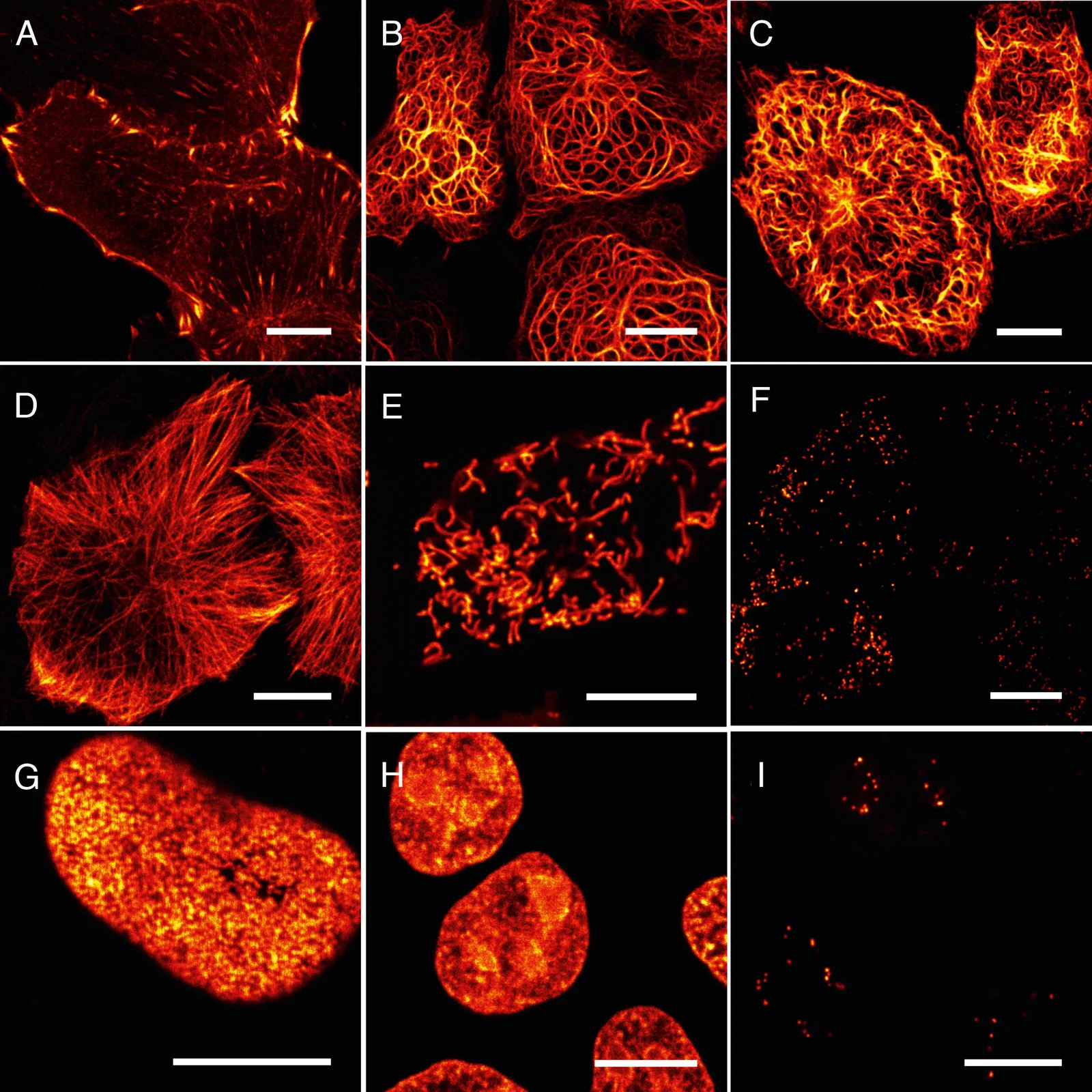
Confocal images of living HeLa cells expressing elite-niRFP704 fusion proteins. The cells were incubated for 2 h with 25 µM BV prior to imaging. (*A*) Actin, elite-niRFP704-actin; (*B*) Keratin, keratin-elite-niRFP704; (*C*) Vimentin, vimentin-elite-niRFP704; (*D*) Microtubules, elite-niRFP704-Map2; (*E*) Mitochondria, Cox8-presequence-elite-niRFP704; (*F*) Peroxisomes, elite-niRFP704-peroxisomal targeting sequence (PTS); (*G*) Nuclear pore complexes, elite-niRFP704-Nup50; (*H*) Nucleus, elite-niRFP704-HIST1H2bn; (*I*) Centromeres, elite-niRFP704-CENPC1. (Scale bar, 10 µm.)

Next, we aimed at exploring elite-niRFP704 for NIR live-cell microscopy and thus excited it at 660 nm. To first investigate the suitability of the elite-niRFP704 as a fusion tag in NIR live-cell microscopy, we cloned several fusion constructs targeting different cellular structures (Fig. 2). We used it to highlight various cytoskeletal elements by expressing translational fusions of elite-niRFP704 with actin (Fig. 2*A*), keratin (Fig. 2*B*), vimentin (Fig. 2*C*), and the microtubule binding protein Map2 (Fig. 2*D*). It was also targeted to the mitochondrial matrix (Fig. 2*E*) and peroxisomes (Fig. 2*F*). In addition, it was fused to the nuclear pore protein Nup50 (Fig. 2*G*), the DNA binding protein histone2 (Fig. 2*H*), as well as to the centromeric protein CENPC11 (Fig. 2*I*). elite-niRFP704 behaved well in all tested constructs and was targeted to the correct subcellular localizations. We conclude that elite-niRFP704 is a versatile tag for live-cell microscopy.

### NIR STED Nanoscopy with elite-niRFP704.

**Fig. 3.**
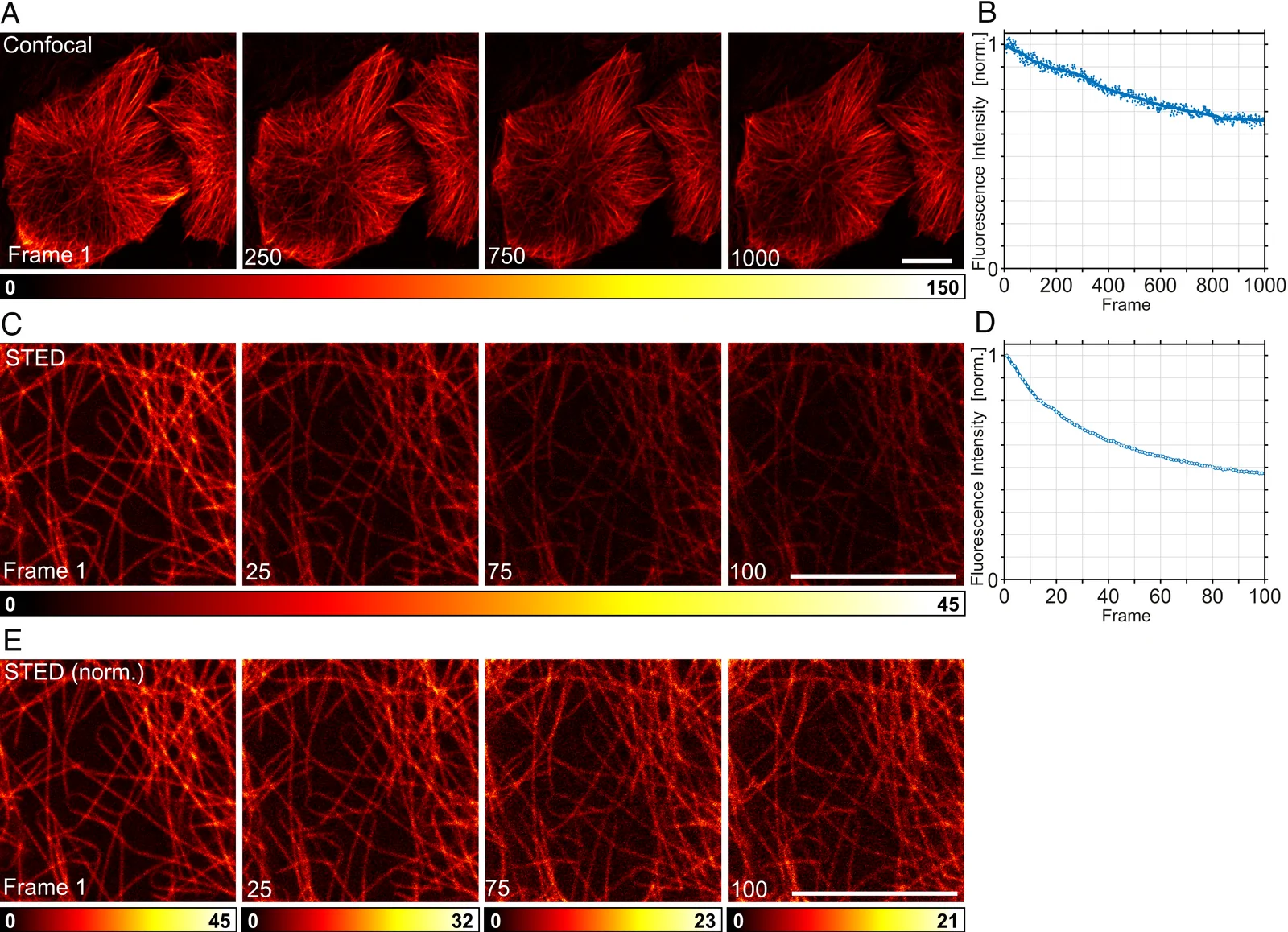
Imaging of HeLa cells transiently expressing elite-niRFP704-Map2. (*A*) Recording of 1,000 consecutive confocal images. (*B*) Average brightness per frame during 1,000 consecutive confocal images as shown in (*A*). (*C*) Recording of 100 consecutive STED images. (*D*) Average brightness per frame of STED images as shown in (*C*). (*E*) STED images from (*C*) with color scales normalized to the average brightness. (Scale bar, 10 µm.)

In order to analyze the photostability of elite-niRFP704 in NIR live-cell imaging, we expressed a fusion of elite-niRFP704 with the microtubule binding protein Map2 in human cells and recorded 1,000 consecutive frames on living cells with a confocal microscope using an excitation wavelength of 660 nm (Fig. 3*A*). During this recording period, the overall fluorescence was reduced by only ~35% and no signs of phototoxicity were apparent (Fig. 3*B*). We conclude that elite-niRFP704 is very photostable and long recording times are well tolerated. This was a good indication that elite-niRFP704 could be used as a label for NIR STED imaging.

To determine the optimal STED wavelength for elite-niRFP704, we examined the photobleaching and the re-excitation by STED light, as well as the STED efficiency for STED wavelengths of 790 to 840 nm (*SI Appendix*, Fig. S2) at an excitation wavelength of 660 nm. We found that a STED wavelength of 820 nm offered the best compromise between low saturation intensity of excited state depletion, low photobleaching, and low re-excitation from the ground state.

In NIR STED imaging (excitation; 660 nm; depletion: 820 nm), we recorded 100 frames (Fig. 3*C*) of cells transiently expressing elite-niRFP704-Map2. After 100 images, only about 50% of the fluorescence signal was lost (Fig. 3*D*) and fine structural details of the dynamic microtubule structures were still visible (Fig. 3*E*).

**Fig. 4.**
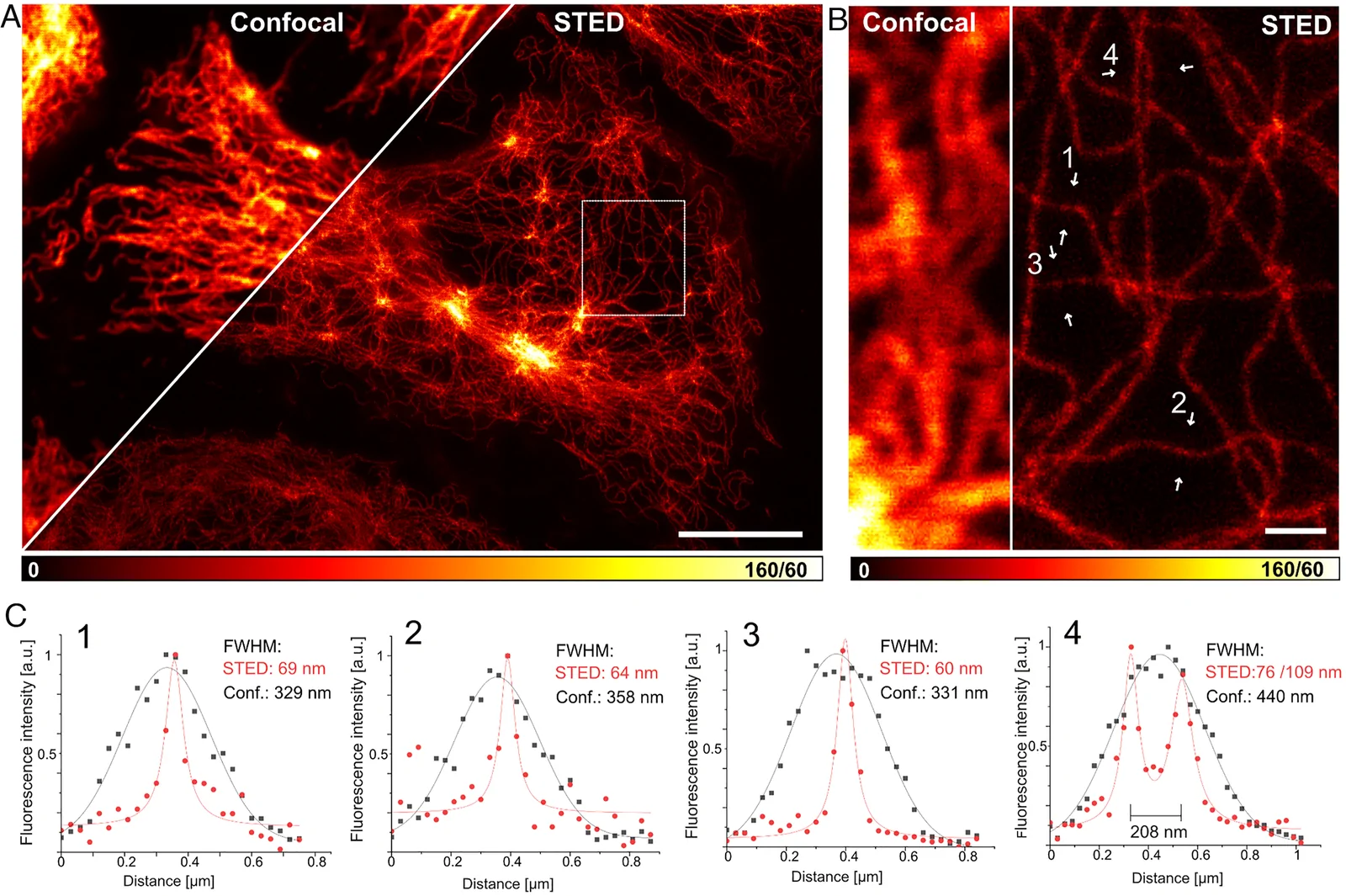
STED superresolution microscopy of endogenously tagged living U2OS cells expressing vimentin-elite-niRFP704. 2 h before imaging the cells were incubated with 25 µM BV. (*A*) Confocal and STED overview image; (*B*) Magnification of marked region from overview image. (*C*) Line profiles taken at the positions indicated in (*B*). [Scale bars, 10 µm (*A*) and 1 µm (*B*).]

In order to investigate the use of elite-niRFP704 at endogenous expression levels for NIR STED superresolution microscopy, we created a genome edited human U2OS cell line expressing vimentin-elite-niRFP704 from the native genomic locus. In confocal recordings, the vimentin filaments had a Full Width at Half Maximum (FWHM) of about 300 nm, corresponding to the diffraction limit (Fig. 4). In NIR STED recordings, the vimentin filaments were imaged about four times sharper with a FWHM of below 70 nm. Also, NIR STED imaging of transiently transfected HeLa cells expressing elite-niRFP704 fused to Nup50 (*SI Appendix*, Fig. S3 *A* and *B*) and keratin (*SI Appendix*, Fig S3 *C* and *D*), respectively, revealed fine details that were indiscernible in the confocal images. As addition of 25 µM BV for 2 h to the cell culture improved the signal-to-noise ratio without introducing any obvious negative effects, we routinely added BV prior to imaging. Nevertheless, endogenous BV levels in untreated HeLa cells were sufficient to enable NIR STED superresolution imaging of living cells expressing a keratin-elite-niRFP704 fusion protein without exogenous BV supplementation (*SI Appendix*, Fig. S4 *A* and *B*). A stronger effect of BV addition was observed in transiently transfected cells (*SI Appendix*, Fig. S4*C*).

### Two-Channel Live-Cell NIR-FLIM.

The difference in fluorescence lifetime between elite-niRFP704 and its spectrally overlapping template miRFP703 is 0.43 ns (Fig. 1*D*). We reasoned that this difference should be sufficient to separate the two proteins solely based on their fluorescence lifetime and thus to enable two-color NIR FLIM.

**Fig. 5.**
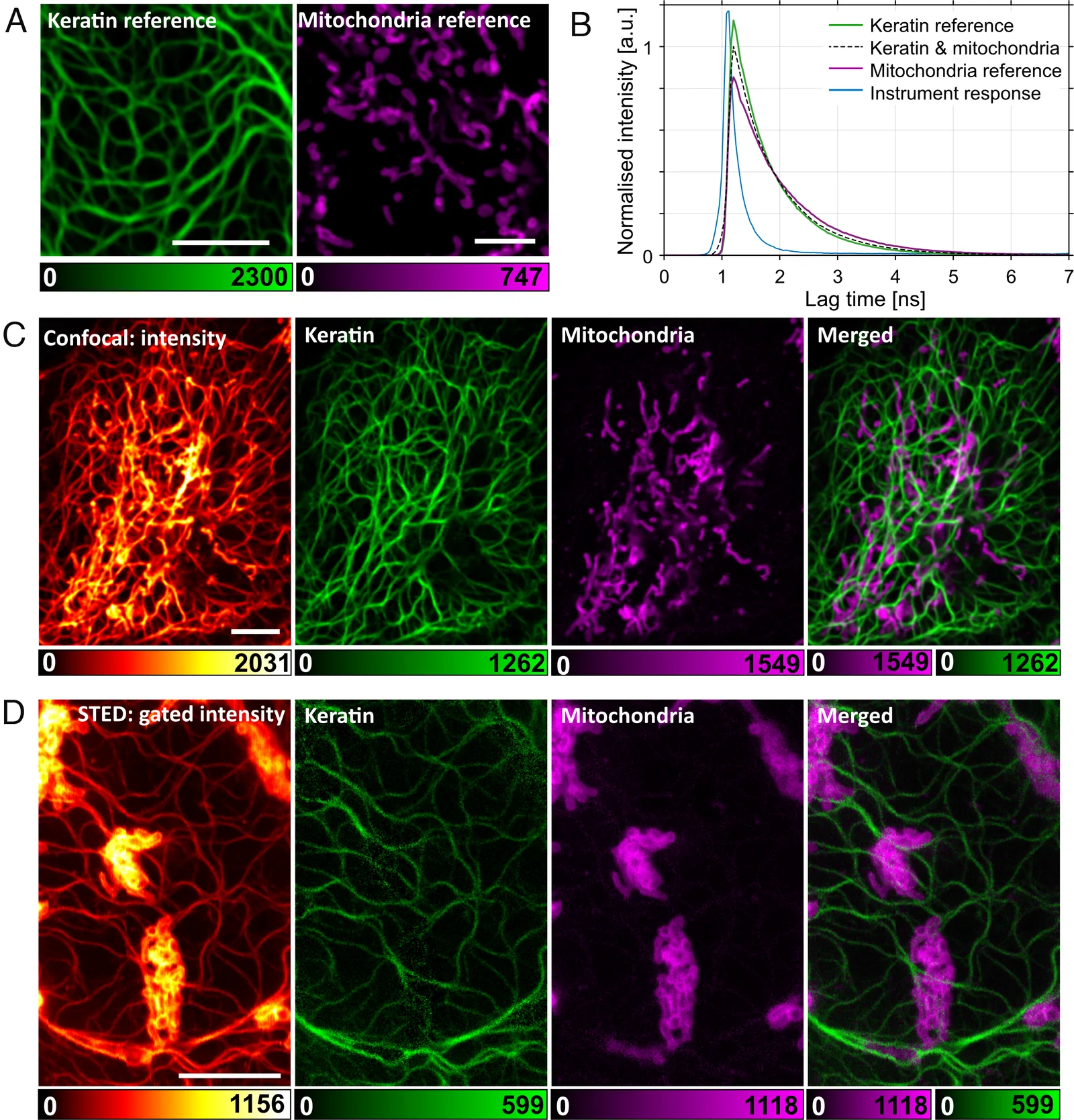
FLIM in the NIR region. (*A*) Reference images of keratin-miRFP703 (*Left*) and elite-niRFP704-Tomm20 (*Right*). (*B*) Instrument response function (FWHM ≈ 200 ps) and fluorescence lifetime histograms of keratin reference, mitochondria reference, and keratin together with mitochondria. The histograms were accumulated by summing the histograms of all pixels in FLIM images of cells expressing only keratin-elite-niRFP704 or only elite-niRFP704-Tomm20 or both keratin-elite-niRFP704 and elite-niRFP704-Tomm20, respectively. (*C*) from left to right: Fluorescence intensity of the confocal fluorescence lifetime image with HeLa cells expressing keratin-miRFP703 and elite-niRFP704-Tomm20; decomposed keratin channel; decomposed Tomm20 channel; and merged image. (*D*) from left to right: Fluorescence intensity of the STED fluorescence lifetime image with HeLa cells expressing keratin-miRFP703 and elite-niRFP704-Tomm20; decomposed keratin channel; decomposed Tomm20 channel; and merged image. Color scales: 0 to 99.9% percentile. (Scale bar, 5 µm.)

To establish two-color NIR FLIM, a cell line expressing keratin-miRFP703 from a genomic locus was transfected with an expression plasmid encoding elite-niRFP704-Tomm20. For NIR FLIM, fluorescence was excited at 660 nm and time-resolved fluorescence was detected from 680 to 750 nm using a superconducting nanowire single photon detector (SNSPD) (Single-Quantum, Delft, The Netherlands) connected with a TCSPC system (SPC-150, Becker & Hickl, Berlin, Germany). A maximum likelihood decomposition of the FLIM lifetime histograms cleanly separated the two different species (Fig. 5 *A* and *B*). The performance of the algorithm was evaluated and compared to a simple least squares decomposition using simulated data (*SI Appendix*, Fig. S5) and images from cells expressing only one of the two fluorophores (Fig. 5 *A*–*C*). We found that by weak Gaussian image smoothing, only about 2% of the counts were misattributed (Fig. 5*A* and *SI Appendix*, Table T2). Hence, using maximum likelihood decomposition, miRFP703 and elite-niRFP704 were faithfully separated and assigned to two channels in confocal and STED imaging. In the confocal mode, FLIM crosstalk was negligible in the images (Fig. 5 *A*–*C*). In STED FLIM, minor crosstalk was visible, in particular at sites where the rather low fluorescence signal from keratin was masked by overlapping bright mitochondria (Fig. 5*D*). However, the separation of the two structures was distinct without any apparent loss of resolution. Hence, we demonstrated two-channel live-cell confocal and STED imaging entirely in the NIR spectral region using miRFP703 and elite-niRFP704 fusion proteins.

## Discussion

Using fluorescence lifetime as a selection criterion during bacterial screening, we engineered elite-niRFP704, a bright monomeric fluorescent protein optimized for extended live-cell STED imaging. Previous efforts by us and others led to the development of NIR fluorescent proteins compatible with live-cell STED imaging (17, 18). elite-niRFP704 exhibits the longest fluorescence lifetime and the highest fluorescence quantum yield reported thus far for a STED-compatible bacterial phytochrome-derived NIR fluorescent protein. The molecular brightness of elite-niRFP704 has been doubled in comparison to its template miRFP703.

Our findings suggest that the mutation I253S plays a central role in modulating the photophysical properties of elite-niRFP704. The position 253 is located in close proximity to the binding chromophore. The closely related fluorescent protein miRFP670 has a cysteine at this position causing a substantial increase in fluorescence quantum yield while inducing a blue shift in the spectral properties, relative to miRFP703 (21). This effect could be explained by generation of an alternative covalent chromophore attachment site in miRFP670 (23, 30, 31), as compared to miRFP703 which exhibits a more constrained thioether linkage between the protein and the BV-chromophore (32). Additionally, it was shown that the π–electron conjugation to BV’s A-ring is broken in miRFP670 causing the blue-shift (32).

In elite-niRFP704, the I253S substitution is unlikely to form an analogous covalent linkage because the hydroxyl group of serine is considerably less reactive than the thiol group of cysteine. Instead, we propose that replacement of the hydrophobic isoleucine residue with a polar serine alters the local chromophore environment through modified noncovalent interactions, including changes in polarity, hydrogen-bonding networks, and chromophore packing. Such local remodeling of the BV-binding pocket may explain both the emergence of spectrally distinct states and the improved photophysical properties observed in elite-niRFP704.

Importantly, this spectral heterogeneity does not appear to compromise the practical value of the protein. Rather, the combined effect of I253S and the additional introduced mutations results in favorable ensemble properties, including increased quantum yield, prolonged fluorescence lifetime, enhanced brightness, and improved photostability, all of which directly translate into superior STED imaging performance.

elite-niRFP704 substantially increased resistance to photobleaching during live-cell imaging, extending imaging duration to more than 100 successive STED frames before signal intensity decreased by 50%. In addition, the protein enabled imaging of vimentin filaments with spatial resolutions below 70 nm, representing an approximately fourfold improvement over diffraction-limited imaging while requiring lower depletion laser powers.

Recent advances in rationally engineering new fluorescent proteins using lifetime-based screening (24, 27) have created a broad set of proteins aimed at FLIM-STED imaging, albeit in the visible spectrum. elite-niRFP704 extends this toolbox further into the NIR spectral range for FLIM-STED using fluorescent proteins. Using the prolonged lifetime of elite-niRFP704 in combination with its spectrally similar template, it was possible to perform two-channel live-cell NIR-STED FLIM imaging with minimal crosstalk.

One potential limitation of elite-niRFP704 is that efficient depletion requires an 820-nm STED wavelength, which lies further into the NIR region than most commercially available depletion lasers. This may be an obstacle for its immediate use with commercial systems. Even so, in light of recent advances in in vivo imaging (33), coupled with successful attempts of live tissue STED imaging with fluorescent proteins (34, 35), and development of new NIR-FPs (36), elite-niRFP704 is likely to be valuable for multichannel NIR STED imaging in living tissues, where scattering and absorption at shorter wavelengths is of particular concern.

## Methods

### Bacterial Expression and Mutagenesis.

For expression of bacterial phytochromes in *E. coli*, the respective coding sequences were cloned into a pBad expression plasmid coexpressing heme oxygenase-1 (Addgene plasmid #80341). To this end, the respective coding sequences were amplified via PCR (primers listed in *SI Appendix*, Table T1). Subsequently, the PCR products were digested with BglII and EcoRI and ligated into the BamHI and EcoRI digested expression plasmid. Error-Prone mutagenesis (37), site-directed mutagenesis (38), and multiple-site mutagenesis (39) were performed using standard protocols with modifications as described in (40, 41). Residue positions 9, 16, 17, 22, 23, 25, 43, 47, 106, 127, 155, 168, 170, 179, 180, 184, 192, 197, 200, 201, 202, 203, 205, 206, 207, 210, 211, 216, 230, 248, 249, 250, 251, 253, 254, 257, 258, 260, 261, 264, 266, 268, 274, 280, 281, 282, 284, 290, 292, 300, 301, 305, 311 were subjected to mutations via error-prone PCR.

### Fluorescence Lifetime Screening.

For selection of variants with increased fluorescence lifetime and brightness, a confocal fluorescence microscope (DM6000, Leica Microsystems, Wetzlar, Germany) was customized and automated. For excitation of the fluorescent proteins, a pulsed 670 nm laser was used (LDH-P-C-670, PicoQuant, Berlin, Germany) at a pulse rate of 20 MHz. The excitation light was focused into the sample using an air objective (N Plan 20×/0.4NA air, Leica Microsystems). NIR light (690 to 740 nm) was collected using the same objective and focused through a pinhole on an avalanche photodiode (MPD-020-CTB, Micro Photon Devices, Bolzano, Italy). Single photons were counted in lifetime histograms via a TCSPC system (SPC-830, Becker&Hickl, Berlin, Germany). The microscope was controlled via custom LabVIEW (National Instruments, Austin, TX) software and the fluorescence lifetime data were analyzed and fitted with custom MATLAB (The Mathworks, Natick, MA) software based on a method described in (42).

### Protein Expression in *E. coli* and Purification.

For protein expression, the respective protein encoded on a pBad expression plasmid was transformed into *E. coli* strain BL21-AI (Invitrogen, Carlsbad, CA). Bacterial cells were grown on agar plates or in liquid culture containing LB medium supplemented with 50 µg/mL ampicillin and 0.02% arabinose for induction of expression. Protein expression was performed at 37 °C for 18 to 24 h.

The protein purification was performed by Ni-NTA affinity chromatography (His SpinTrap Kit, GE Healthcare, Little Chalfont, Great Britain) according to the manufacturer’s instructions. Protein concentration either was measured spectroscopically via protein absorption at 280 nm or with a Bradford assay. After purification, purified protein was taken up in 100 mM Tris-HCl, 150 mM NaCl, pH 7.5.

### Spectral Measurements.

All spectra were measured with purified protein in 100 mM Tris-HCl, 150 mM NaCl, pH 7.5. Absorption spectra were measured using a Varian Carry 4,000 UV/vis spectrometer. The extinction coefficient of elite-niRFP704 at 655 nm was calculated relative to the Soret maximum at 388 nm, which corresponds to biliverdin absorption with a reported extinction coefficient of 39,900 M^–1^ cm^–1^ (10, 28). Excitation and emission spectra were measured with a Varian Eclipse fluorescence spectrometer. The fluorescence lifetime was measured with a Quantaurus-Tau fluorescence lifetime spectrometer. The fluorescence quantum yield was determined using a plasmonic nanocavity (29).

### pH Spectra.

Fluorescence excitation spectra at different pH values for determination of p*K*_a_ value were measured using a Cytation 3 plate reader (BioTek, Winooski, VT) with fluorescence excitation at 640 to 660 nm and detection at 690 to 710 nm wavelength at a protein concentration of 0.1 µg/µL. All measurements were performed in replicates of at least n = 3. The values of each measurement were normalized to the corresponding maximal signal. The p*K*_a_ value was determined by fitting the data to a monophasic model. Absorption spectra at different pH values were measured using a Varian Cary 4,000 UV/Vis spectrometer. Excitation and emission spectra of the same samples were measured with a Varian Eclipse fluorescence spectrometer. For the different pH conditions, the following buffers were prepared:

1. pH 3 to 5.75: 100 mM citric acid, 150 mM NaCl
2. pH 6 to 7: 100 mM KH_2_PO_4_, 150 mM NaCl
3. pH 7.5 to 8.5: 100 mM Tris, 150 mM NaCl
4. pH 9 to 9.5: 100 mM glycine, 150 mM NaCl

### FLIM.

FLIM measurements were performed with a scanning fluorescence microscope (Abberior Instruments, Göttingen, Germany) that was also used for confocal and STED measurements. The fluorescence photons were detected with a prototype superconducting nanowire single-photon detector (43) kindly provided by Single Quantum and Delft University of Technology (Delft, The Netherlands) in combination with a TCSPC system (SPC-150, Becker&Hickl, Berlin, Germany).

The analysis of the FLIM images was performed with custom Python software. The lifetime histogram of each pixel was analyzed individually. The measured fluorescence decay histogram was decomposed into the most likely linear combination of the measured characteristic histograms assuming Poisson noise (42). The nanowire detector featured a timing jitter of about 120 ps full width at half-maximum (FWHM) and a quantum yield of about 45% in the NIR spectral range when feeding the signal through a 2 m long Ø50 μm graded-index fiber. Including the excitation pulse duration, the instrument response function was measured at about 200 ps FWHM (Fig. 5*B*). As the timing did not modify with the detection count rate, the reference histograms were applied without temporal scaling or shifting. Furthermore, the detector dark count rate was so insignificant that it was ignored.

Given the composite lifetime histogram $h\left({\tau_{i}\leq \tau <\tau }_{i+1}\right)=h_{i}$ measured at a sample position for the time bins i and the reference histograms $r_{j}\left({\tau_{i}\leq \tau <\tau }_{i+1}\right)=r_{j,i}$ normalized to $\sum_{i}r_{j,i}=1$, the decomposition finds the counts ${\hat{c}}_{j}$ emitted by the species j such that the estimated histogram ${\hat{h}}_{i}=\sum_{j}r_{j,i}{\hat{c}}_{j}$ best matches the measured histogram. We used a least squares QR solver to find the initial counts by matrix division $\left[c_{j}\right]=\left[r_{j,i}\right]\backslash \left[h_{i}\right]$. Negative counts were zeroed and the other counts recalculated in the same way by excluding the references that led to negative counts.

The counts in each bin of the measured histogram contain shot noise, that is the counts h are Poisson distributed around $\hat{h}$:

[1] $$\text{Pr}\left\{h\;=\;\hat{h}\right\}\;=\;\frac{{\hat{h}}^{h}\text{exp}\left(-\hat{h}\right)}{h!}.$$

We refined the counts by maximizing the log-likelihood

[2] $$\begin{array}{c} \text{ln}\prod_{i}\text{Pr}\left\{h_{i}\;=\;{\hat{h}}_{i}\right\}\;=\;\sum_{i}\left(h_{i}\;\text{ln}\;{\hat{h}}_{i}\;-\;{\hat{h}}_{i}\;-\;\text{ln}\;\left(h_{i}!\right)\right)\;\\ \;=\;\sum_{i}h_{i}\;\text{ln}\;{\hat{h}}_{i}\;-\sum_{i}\;{\hat{h}}_{i}\;-\sum_{i}\text{ln}\left(h_{i}!\right). \end{array}$$

that is by minimizing the variable part of the negative log-likelihood:

[3] $$\underset{{\hat{c}}_{j}}{\text{min}}\left(\sum_{i}{\hat{h}}_{i}\;-\;\sum_{i}h_{i}\;\text{max}\left(-50,\text{ln}\;{\hat{h}}_{i}\right)\right).$$

We truncated $\ln\;{\hat{h}}_{i}$ at -50 because near-zero estimates would dominate the log-likelihood otherwise. In addition, nonnegative constraints on the counts ${\hat{c}}_{j}$ were implemented by searching for their square roots.

### Constructs for Expression in Mammalian Cells.

Primers used for amplifying specific sequences are listed in *SI Appendix*, Table T1.

To target actin, the coding sequence of actin was amplified from pTagFP635-actin (Evrogen, Moscow, Russia). Thereafter, the coding sequence of human alpha-tubulin in pEGFP-Tub (BD, Biosciences Clontech, Franklin Lakes, NJ) was exchanged for actin via BamHI and XhoI. The final fusion construct of actin with elite-niRFP704 was produced by swapping EGFP via NheI and BglII for elite-niRFP704.

In case of CENPC1, HIST1H2bn, and peroxisomal targeting, the coding sequence of elite-niRFP704 was amplified using the listed primers (*SI Appendix*, Table T1). The coding sequence of CENPC1 was obtained from pDORN233-CENP-C29 (Human ORFeom Collection) and was swapped for the coding sequence of alpha-tubulin in the construct pEGFP-Tub (BD, Biosciences Clontech) as described (18). Thereafter, EGFP was exchanged for elite-niRFP704 via NheI and XhoI.

For HIST1H2bn, EGFP was swapped for elite-niRFP704 via NheI and BglII in the construct pEGFP- HIST1H2bn, which was described by (18). To produce the C-terminal fusion construct of the PTS with elite-niRFP704, EGFP was swapped for elite-niRFP704 in the construct pEGFP-PTS described by (18).

To target the mitochondrial matrix, DsRed in pDsRed1-Mito (BD Biosciences Clontech, Franklin Lakes, NJ) was swapped via AgeI and NotI with the PCR product of elite-niRFP704.

To generate a fusion construct of keratin-18, the coding sequence of elite-niRFP704 was amplified and was swapped with the TagRFP coding sequence in pTagRFP-Keratin18 (Evrogen) using KpnI and NotI, resulting in pKrt18-elite-niRFP704.

For Map2 and Nup50, the coding sequence of elite-niRFP704 was amplified via PCR. To generate a Map2-elite-niRFP704 fusion construct, Map2 was amplified as described (40) and the PCR fragment was cloned into pEGFP-tub (BD, Biosciences Clontech) via XhoI and BamHI. Subsequently, we exchanged EGFP for the elite-niRFP704 PCR product via BglII and NheI. The Nup50 fusion construct was cloned by swapping mEmerald for the elite-niRFP704 PCR product via NheI and BglII in pmEmerald–Nup50 construct (Addgene #54209).

To target vimentin, the coding sequence of elite-niRFP704 was amplified and swapped for mKate2 via AgeI and NotI in the vector pmKate2-vimentin (Evrogen).

The outer mitochondrial membrane was targeted by an elite-niRFP704-Tomm20 fusion construct. To this end, the coding sequence of Tomm20 was amplified from pDONR223-Tomm20 (Human ORFeom Collection, gene id 9804) and swapped via AgeI and NheI for the Mito targeting sequence in pDsRed1-Mito (Clontech Laboratories, Mountain View, CA). DsRed was subsequently exchanged via AgeI and NotI for the elite-niRFP704 coding sequence.

### Construction of Genome Edited Mammalian Cell Lines.

For endogenously tagging of vimentin, the coding sequence of elite-niRFP704 was amplified and cloned via NcoI and NotI into the pUC57-Vim donor plasmid described by Ratz et al. (44). Subsequently, U2OS cells were transfected with the bicistronic nuclease plasmid (44) and the corresponding donor plasmid using a TurboFect Kit (Thermo Fisher Scientific, Waltham, MA). Approximately 7 d after transfection, single cells showing fluorescence were sorted into 96 well plates using fluorescence activated cell sorting (FACS). Within 2 to 3 wk after single cell sorting, the wells were inspected by fluorescence microscopy (Lionheart FX Automated Microscope, BioTek). Wells containing only cells exhibiting the expected vimentin structure were isolated and expanded for further experiments.

For the creation of a landing pad cell (45) line expressing Keratin-elite-niRFP704, a HeLa cell line with a CAG promoter and a Bxb1 attP site in the AAVS1 locus was used. For integration into the Bxb1 attP site, 1 μg of the integration plasmid was cotransfected with 1 μg of the plasmid pCAG-NLS-HA-Bxb1 (46). The plasmid pCAG-NLS-HA-Bxb1 was a gift from Pawel Pelczar (Addgene #51271) and encoded for the transient expression of the Bxb1 recombinase.

### Cell Culture.

HeLa and U2OS cells were transfected with the respective plasmid using the TurboFect Kit (Thermo Fisher Scientific, Waltham, MA). Cells were cultivated in Dulbecco’s modified Eagle’s Medium (DMEM-Medium: 4.5 g/L glucose, GlutaMAX, phenol red, 10% vol/vol FCS, 1 mM sodium pyruvate, 100 µg/mL streptomycin, 100 µg/mL penicillin) either on coverslips (for imaging) or without coverslips in six well plates at 37 °C in a humidified 5% CO_2_ atmosphere. If not stated differently, 25 µM BV was added to the medium approximately 2 h before imaging.

### Seminative Polyacrylamide Gel.

2 to 4 µg purified protein solution in 100 mM Tris-HCl, 150 mM NaCl, 10% sucrose, pH 7.5 was loaded onto a 15% polyacrylamide gel containing 0.1% sodium dodecyl sulfate. As a standard, monomeric protein miRFP703 and dimeric protein iRFP702 were used. The gel was run for 1 h at 20 mA. Subsequently, the NIR fluorescence was detected using a gel imager (GE Healthcare, Chicago, IL) equipped with a 630 nm excitation and a Cy5 filter set.

### Size Exclusion Chromatography.

An Äkta pure chromatography system equipped with a Superdex 200 Increase 10/300 column (GE Healthcare) was used for size exclusion chromatography. The purified protein solution was filtered using 0.2 µm spin columns (Sartorius, Göttingen, Germany). 250 µL at a concentration of 10 µM was added to the column and eluted at a flow rate of 0.75 mL/min with 100 mM Tris-HCl, 150 mM NaCl, pH 7.5. The UV monitor U9-L (GE Healthcare) at 280 nm was used for protein detection. miRFP703 and iRFP702 were employed for comparison to template proteins. For comparison with sizing standards (protein standard mix, Merck, Darmstadt, Germany), a volume of 100µL was injected at a concentration of 1 mg/mL and eluted at a flow rate of 0.7 mL/min. All runs were performed at 6 °C.

### FACS Measurements.

FACS measurements were performed using a modified FACS system (BD influx™ cell sorter, BD Biosciences, Franklin Lakes, NJ) with a 671 nm laser (SDL-671-300 T, Shanghai Dream Laser, Shanghai, China) for NIR excitation. The detection windows for near-infrared fluorescence were set at 690 to 720 nm and 720 to 766 nm. 25 µM BV was added to the cells approximately 2 h before FACS measurements. Immediately before the measurements, cells were trypsinized and resuspended in PBS.

### NIR Imaging.

Imaging was performed approximately 24 h after transfection in HDMEM (HEPES buffered DMEM) without the pH indicator phenol red. If not stated differently, 25 µM BV was added to the cells 2 h before imaging. Confocal, STED, and FLIM imaging was performed on a modified scanning fluorescence microscope (Abberior Instruments) using an Olympus microscope body (IX83, Olympus, Tokyo, Japan). The microscope was equipped with a titanium-sapphire laser (MaiTai, Spectra-Physics, Santa Clara, CA; pulse rate: 79 MHz, output pulse duration ~150 fs) to generate both the excitation and STED beams. To this end, the laser beam from the titanium-sapphire laser (mode locked at 790 to 840 nm wavelength) was split into two beams by a polarizing beam splitter. One beam was fed into a crystal fiber (Femto WHITE 800, NKT Photonics, Birkerød, Denmark) to generate a white light spectrum. Using a tunable band pass filter (VersaChrome HC 708/13, AHF Analysetechnik, Tübingen, Germany) light of 645 to 665 nm wavelength was selected from the white light spectrum and used as excitation light (*SI Appendix*, Fig. S6). The other beam’s pulses were stretched with glass rods and used for STED. To focus the beams into the sample and to collect the fluorescence light, an oil-immersion objective was used (UPlanSApo 100×/1.4NA oil, Olympus). The NIR fluorescence light (680 to 750 nm) was focused through a pinhole on a fiber-coupled single-photon counting module (SPCM-AQRH-13-FC, Excelitas Technologies, Waltham, MA) for confocal and STED imaging and on a Ø50 μm graded-index fiber-coupled customized superconducting nanowire detector (Single Quantum and Delft University of Technology, Delft, The Netherlands) for FLIM and STED-FLIM imaging (29). All laser powers were determined using a power meter (PM200, Thorlabs, Newton, NJ) with a microscope slide power sensor (S175C, Thorlabs).

The intensity line profiles were averaged across three adjacent pixels at the indicated sites. Data points were fitted with a Lorentzian (STED) or Gaussian (confocal) function.

## Supplementary Material

Appendix 01 (PDF)

## Data Availability

Data used in the study are available at Zenodo (https://doi.org/10.5281/zenodo.21623096) (47).
