# Supplementary material for: Bright monomeric fluorescent protein elite-niRFP704 for two-channel near-infrared STED nanoscopy
Source: Proc Natl Acad Sci U S A. 2026 Aug 25;123(35):e2533451123. doi: 10.1073/pnas.2533451123 (PMC13535109; doi:10.1073/pnas.2533451123)
Supplement: Supplementary file 1 [file pnas.2533451123.sapp.pdf]

Supplementary information

Bright monomeric fluorescent protein elite-niRFP704 for two-channel near-infrared STED nanoscopy

Suppl. Table T1 | List of primers used for cloning experiments

**Coding sequence**	**Forward/Reverse Primer**
Pre-screen mutants (in *E. coli*)	AGGGCTCGAGCATGTCGGTACCGCTGACTA/ TTCGAATTCGCTCTCAAGCGCGGTGATCCG
actin	CGTCTCGAGCTATGGATGATGATAT/ CCGGTGGATCCTTACCTAGAAGCATTTGCGG
elite-niRFP704 (for actin) CENCP1, HIST1H2bn, PTS)	ATCCGCTAGCGCTAATGGTAGCAGGTCATGCC/ CTCGAGATCTGAGTCCGGAGCTCTCAAGCGCGGTGAT
elite-niRFP704 (for CENCP1, HIST1H2bn, PTS)	ATCCGCTAGCGCTAATGGTAGCAGGTCATGCC/ CACTCGAGATCTGAGTCCGGAGCTCTCAAGCGCGGTGAT
elite-niRFP704 (for Mito, keratin-18)	CGACCGGTCGCCACCATGGTAGCAGGTCATGCC/ AGCTGTGCGGCCGCTCAGCTCTCAAGCGCGGTGAT
elite-niRFP704 (for Map2, NUP50)	CACTCGAGATCTGAGTCCGGAGCTCTCAAGCGCGGTGA/ TCCGCTAGCGCTACCGGTCGCCACCATGGTAGCAGGTCATGC
elite-niRFP704 (for vimentin)	TCCACCGGTCGCCACCATGGTAGCAGGTCATGCCTCTGGCA/ GTCGCGGCCGCTCAGCTCTCAAGCGCGGT
Tomm20	AGATCCGCTAGCATGGTGGGTCGGAACAGCGCC/ TGGCGACCGGTGGATCCCCTTCCACATCATCTTCAGCC
elite-niRFP704 (for Tomm20)	GGATCCACCGGTCGCCACCATGGTAGCAGGTCATGCCTCT/ TCTAGAGTCGCGGCCGCTAGCTCTCAAGCGCGGTGAT
elite-niRFP704 (for endogenous tagging of vimentin)	ATATTAGCGGCCGCCGGTAGTGGTTCAGGGGTAGCAGGTCATGCCTCTGGC/ TTAATTCCATGGTTAGCTCTCAAGCGCGGTGATCCG

Suppl. Table T2 | Assigned signal fractions upon decomposition of the reference images of Fig. 5a. Prior to the decomposition, the images were smoothed by a Gaussian filter of two pixels standard deviation.

	**Least squares decomposition**		**Maximum likelihood decomposition**	
**Structure**	Keratin	Mitochondria	Keratin	Mitochondria
**Keratin reference**	96.5 %	3.5 %	**98.2 %**	1.8 %
**Mitochondria reference**	4.8 %	95.2 %	1.9 %	**98.1 %**

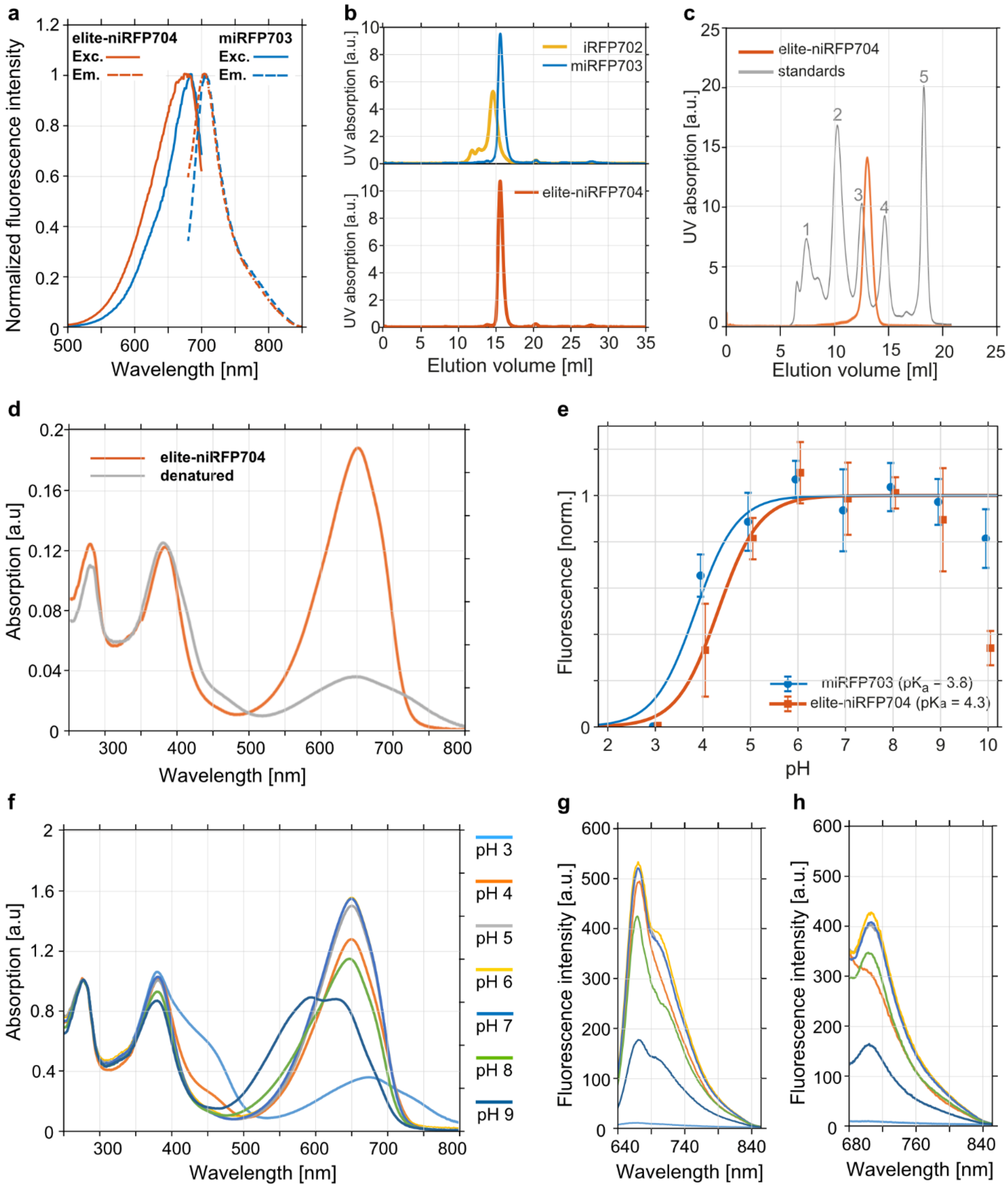

a
elite-niRFP704
Exc.
Em.
miRFP703
Exc.
Em.
Normalized fluorescence intensity
Wavelength [nm]
b
iRFP702
miRFP703
elite-niRFP704
UV absorption [a.u.]
Elution volume [ml]
c
elite-niRFP704
standards
1
2
3
4
5
UV absorption [a.u.]
Elution volume [ml]
d
elite-niRFP704
denatured
Absorption [a.u]
Wavelength [nm]
e
miRFP703 (pK$_a$ = 3.8)
elite-niRFP704 (pKa = 4.3)
Fluorescence [norm.]
pH
f
pH 3
pH 4
pH 5
pH 6
pH 7
pH 8
pH 9
Absorption [a.u]
Wavelength [nm]
g
Fluorescence intensity [a.u.]
Wavelength [nm]
h
Fluorescence intensity [a.u.]
Wavelength [nm]

Suppl. Fig. S1 | Characterization of spectra, oligomeric state and pH stability **a** Normalized excitation and fluorescence spectra of miRFP703 and elite-niRFP704 with red-shifted excitation (670 nm) and emission (680-850 nm); **b** Size exclusion chromatography of precursor proteins and elite-niRFP704; **c** Size exclusion chromatography of elite-niRFP704 along with sizing standards: 1. Bovine thyroglobulin MW ~670,000 Da 2. γ-globulins from bovine blood MW ~150,000 Da 3. Ovalbumin MW ~44,300 Da 4. Ribonuclease A type I-A MW ~13,700 Da 5. p-aminobenzoic acid (pABA) MW ~137 Da; **d** Absorption spectrum of native elite-niRFP704 compared to protein under denaturing conditions (6M guanidine hydrochloride); **e** Stability of fluorescent proteins monitored by their fluorescence intensities at different pH values. The fits ignore the data points at pH 10; **f** Absorption spectra of elite-niRFP704 across different pH values (normalized to the maximum at 278nm); **g** Emission spectra of elite-niRFP704 with a fixed excitation at 630nm across different pH values; **h** Emission spectra of elite-niRFP704 with a fixed excitation at 670 nm across different pH values.

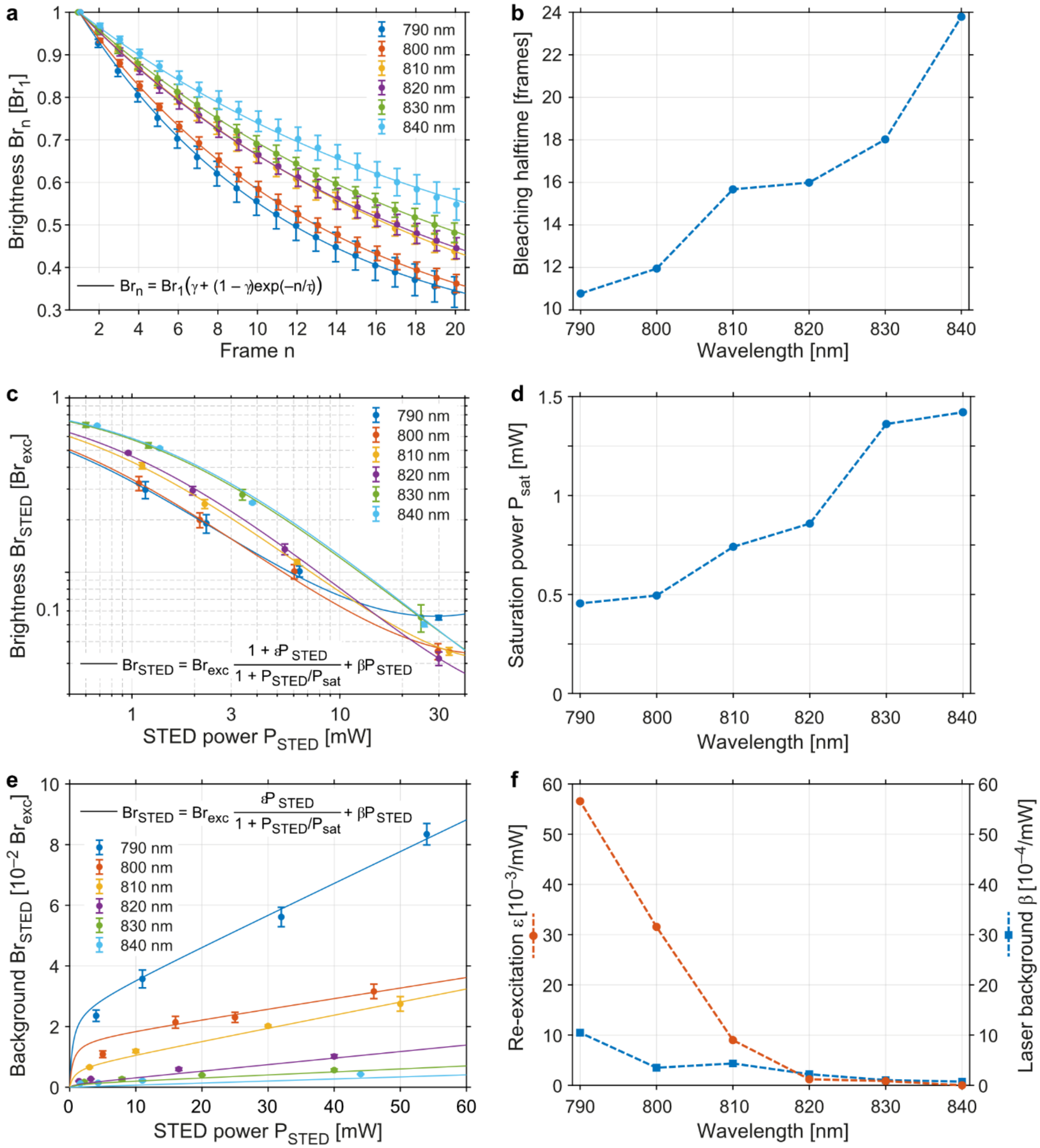

Suppl. Fig. S2 | Characterization of elite-niRFP704 versus STED wavelength and power. **a** Fluorescence intensity versus number of acquired images (inset: STED wavelength). **b** Number of image frames acquired before half of elite-niRFP704 bleached from (**a**). 25μW excitation and 45mW STED laser power (measured at the back aperture of the objective) were used for all conditions in (a) and (b). **c** Mean STED image brightness Br_{STED} over confocal image brightness Br_{exc} versus STED power (inset: STED wavelength). **d** Estimated STED saturation power P_{sat} from (**c**,**e**). **e** Excitation of elite-niRFP704 by the STED light (inset: STED wavelength). **f** Estimated re-excitation rates ε from the ground state and STED laser background β versus wavelength from (**c**,**e**).

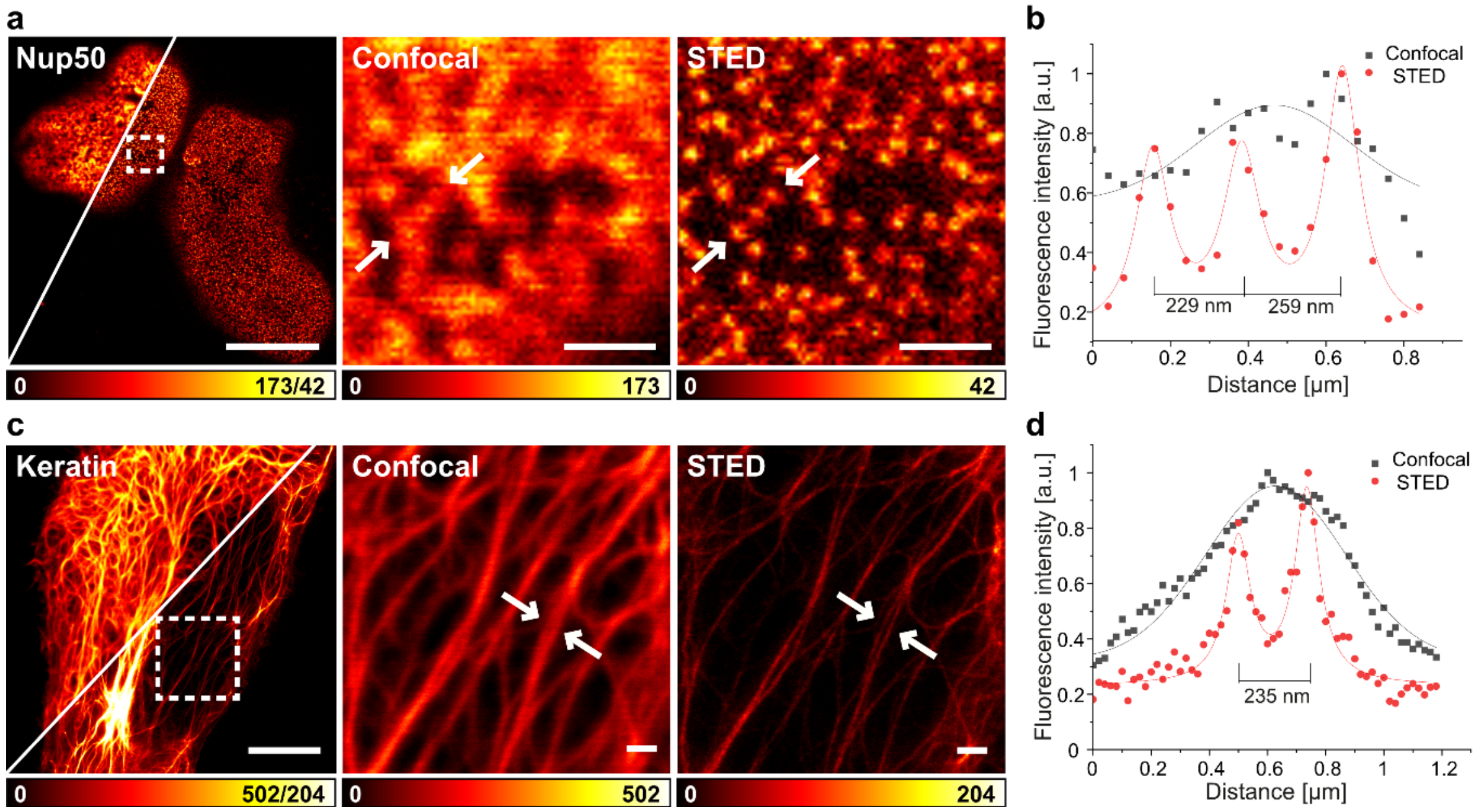

Suppl. Fig. S3 | Live-cell STED super-resolution microscopy using elite-niRFP704. **a** Confocal and STED image of transfected HeLa cells expressing Nup50-elite-niRFP704. The cells were incubated in 25 µM BV approximately 2 h before imaging. **b** Line profiles taken at the position indicated in (**a**). **c** Confocal and STED image of transfected HeLa cells expressing Keratin-elite-niRFP704. **d** Line profiles taken at the positions indicated in (**c**). Scale bars: 10 µm (overviews), 1 µm (magnifications).

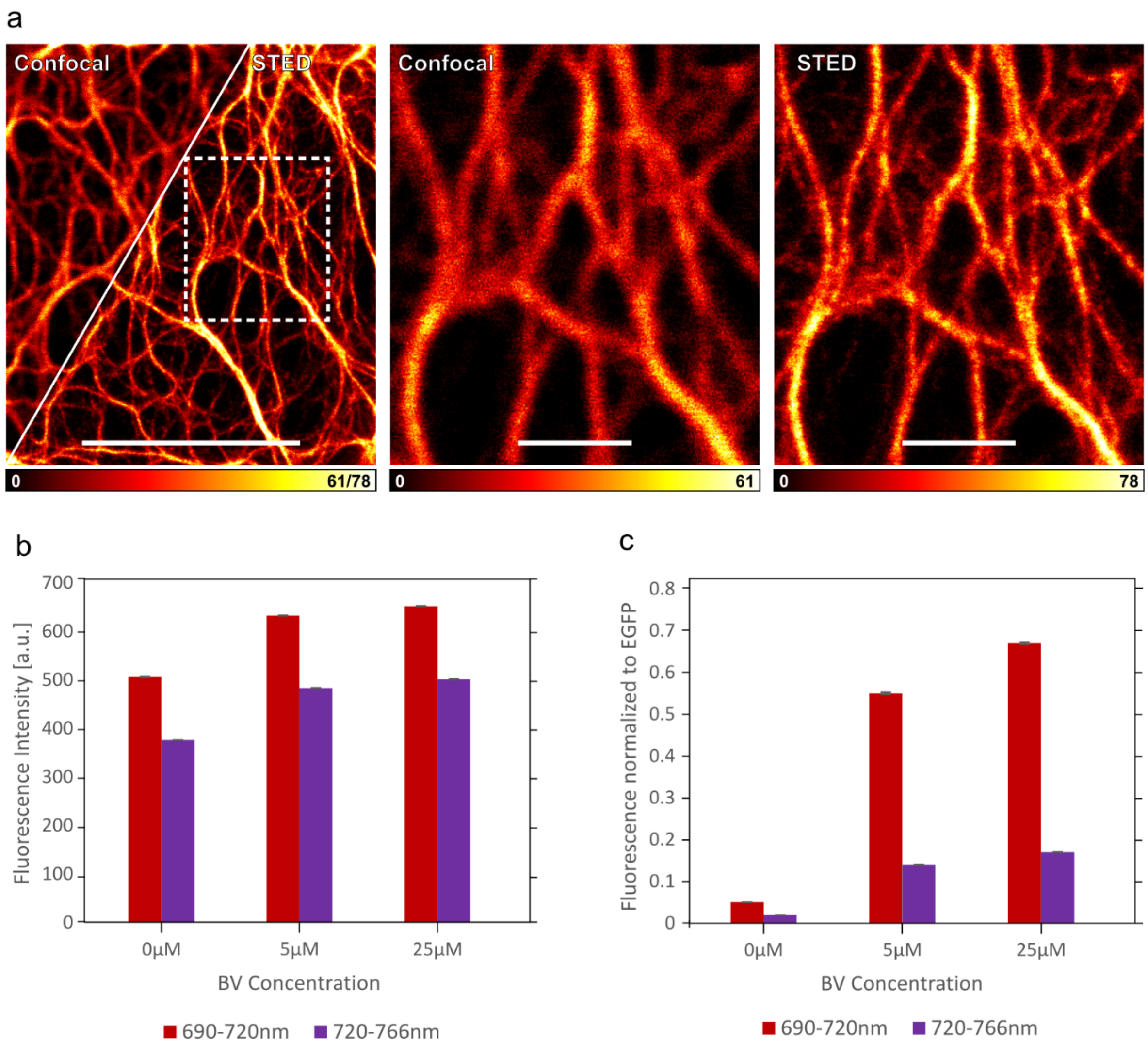

Suppl. Fig. S4 | **a** Live-cell STED super-resolution microscopy of HeLa cell line stably expressing Keratin-elite-niRFP704 without addition of external BV. Scale bars: 10 µm (overview), 2 µm (magnifications); **b** Comparison of flow cytometry data of stable Bxb1 landing pad cell lines expressing the fusion construct keratin-elite-niRFP704-P2A-mEGFP after incubation with 0, 5 and 25 µM BV for 2 h. Fluorescence signal of cells in the detection window between 690 to 720 nm (deep red) and in the detection window between 720 to 766 nm (purple). Black bars indicate standard errors of the measurements. The measurements were performed in replicates of n=3 with 10000 cells for each replicate; **c** Comparison of flow cytometry data of HeLa cells transiently expressing the fusion construct elite-niRFP704-P2A-mEGFP after incubation with 0, 5 and 25 µM BV for 2 h. Fluorescence signal of cells in the detection window between 690 to 720 nm (deep red) and in the detection window between 720 to 766 nm (purple). NIR fluorescence was normalized to mEGFP fluorescence to account for variations in transfection efficiency. Black bars indicate standard errors of the measurements. The measurements were performed in replicates of n=5 with 10000 cells for each replicate.

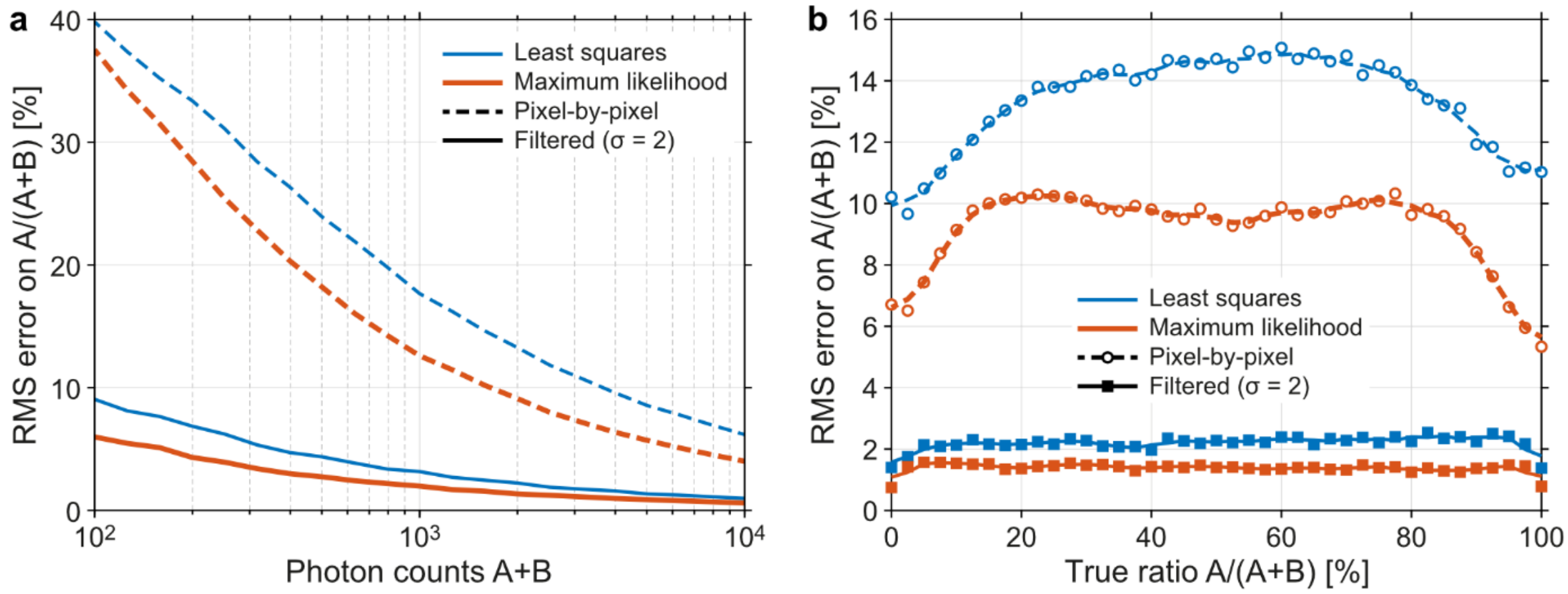

Suppl. Fig. S5 | Validation of the decomposition of the lifetime histograms into the two species. **a** Root-mean-square (RMS) error of the keratin signal A versus photon counts $A+B$ in the lifetime histogram. **b** RMS error of the keratin signal A with respect to the simulated ratio $A/(A+B)$ for photon counts $A+B \approx 2000$. The estimation error is lower at the limits because of the non-negativity constraint. Gaussian filtering of the images (standard deviation $\sigma = 2$ pixels) greatly reduced the RMS error in the confocal images.

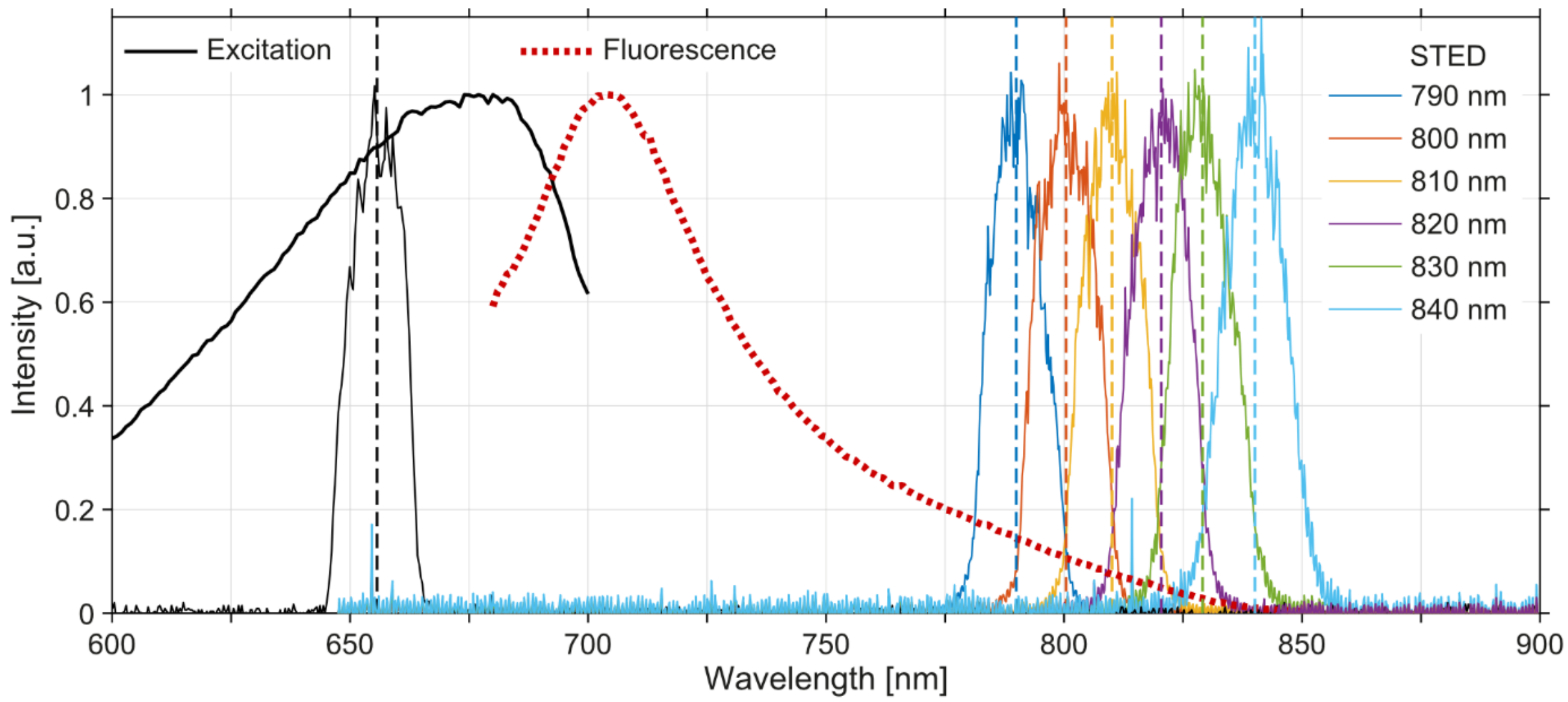

Suppl. Fig. S6 | Excitation and STED laser bandwidth versus the absorption and emission spectrum of elite-niRFP704.
